# Prevalence of cervical infection with HPV type 16 and 18 in Vietnam: implications for vaccine campaign

**DOI:** 10.1186/1471-2407-13-53

**Published:** 2013-02-04

**Authors:** Lan TH Vu, Dieu Bui, Ha TT Le

**Affiliations:** 1 Department of Epidemiology& Biostatistics, Hanoi school of public health, 138 Giang Vo Street, Ba Dinh, Hanoi, Vietnam; 2 National Cancer Institute, 43- Quan Su Street –Hoan Kiem, Hanoi, Vietnam

**Keywords:** Cervical cancer, Human papilloma virus, Vietnam, HPV 16, HPV 18, HPV vaccine

## Abstract

**Background:**

The Expanded Program on Immunization currently considers offering Human Papilomavirus vaccine on a routine basis in Vietnam. However, as the current available vaccine can prevent only two types HPV 16 and 18, before implementing a large-scale vaccine campaign we need information about the prevalence of infection with only HPV 16 and 18 in Viet Nam. This study was done in 5 large cities in Vietnam to estimate the prevalence of HPV 16 and/or 18 infections and to explore the distribution of other high risk types of HPV among married women in these provinces.

**Methods:**

The study employed a cross-sectional design with multistage sampling. The sample size included 4500 married women in two rounds (aged ranged from 18-69 years old, median age: 40 year old). Participant were randomly selected, interviewed and given gynaecological examinations. HPV infection status (by real-time PCR kit using TaqMan probe) and HPV genotyping test (by Reverse dot blot) were done for all participants.

**Results:**

The prevalence of cervical infection with HPV type 16 and/or 18 among married women in this study ranged from 3.1% to 7.4%. Many positive HPV cases (ranged from 24.5% to 56.8%) were infected with other type of high risk HPV which can lead to cervical cancer and cannot prevented by currently available vaccines. In addition to HPV 16 and/or 18, most common types of high risk HPV were types 58, 52, 35 and 45. Awareness about HPV and HPV vaccines was still low in the study samples.

**Discussion:**

While it is relevant to implement an HPV vaccine campaign in Viet Nam, it is important to note that one can be infected with multiple types of HPV. Vaccination does not protected against all type of high risk HPV types. Future vaccine campaigns should openly disclose this information to women receiving vaccines.

**Conclusion:**

High prevalence of infection with HPV high risk types was observed in this study. As HPV infection has a high correlation with cervical cancer, this study emphasizes the need for both primary prevention of cervical cancer with HPV vaccines as well as secondary prevention with screening.

## Background

Human papillomavirus (HPV) is a very common sexually transmitted virus but the infection are often gone without any treatment [1]. However, when the infection persists — in 5% to 10% of infected women — there is high risk of developing precancerous lesions of the cervix, which can progress to invasive cervical cancer. High-risk HPV types are detected in 99% of cervical cancers [2]. In Vietnam, cervical cancer is the most common cause of mortality due to cancer and the second most [3].

Currently, two efficacious prophylatic HPV vaccines are available (Cervarix made by GlaxoSmithKline) protects against only HPV 16 and 18, Gardasil made by Merck protects against HPV 16,18, 6 and 11 [4,5]. In developed countries such as the US, vaccines against HPV were recommended for routine use in females aged 11 to 12 years [4]. In Vietnam, HPV vaccines are not available on a routine use but women can order and pay for it at some preventive health care centers with quite high price (80$per dose x 3 doses). Ministry of Health had approved Cervarix for women from 10–25 year old and Gardasil for women from 9-26 year old. The Expanded Program on Immunization (EPI) currently considers offering HPV vaccine on a routine basis. The current vaccines can protect against 2 high risk HPV types 16 and 18 but previous studies had noted the presence of other high risk types such as HPV 58 in many cases of HPV positive CIN2 and CIN3 [6]. Before making decision to include HPV vaccine into the routine program, it is important to have efficient data on the prevalence and distribution of the vaccine types of HPV (i.e., type 16 and 18) among women.

In the year 2010 and 2011, a survey was conducted in 5 large cities in Vietnam (i.e., Hanoi, Ho Chi Minh, Hue, Can Tho and Thai Nguyen) to collect information on prevalence of HPV cervical infection and risk factors of HPV cervical infection among married women. As reported elsewhere, the prevalence of HPV cervical infection in those cities ranged from 6.1% to 10.2% and the prevalence of high risk HPV infection was from 5.6% to 9.3% [7]. The previous publications however yet provided the prevalence of HPV 16, 18 infection in those city, an important input information for the decision to include HPV vaccines into routine immunization program. This study aims to use data from that survey to provide specific estimation of the prevalence of HPV 16, 18 infections in those cities as well as to examine the distribution of other high risk types of HPV among married women in the 5 big cities of Vietnam.

## Methods

The first round of the study was done in 2010 in two cities, Hanoi and HCM. The estimated sample size for each city was 850 women. Using formula for sample size for a proportion estimate with relative precision, sample size was calculated with the following parameters: anticipated prevalence of HPV as 10%, [8] the relative precision of 22% (The relative uncertainty of the estimated prevalence would be 22% of 10%, about 2.2%), estimated non-respondent rate of 10%. The second round of the study was done in 2011 in three cities, Hue, Can Tho and Thai Nguyen. The estimated sample size for each city was increased to include 1100 women to increase the precision of the estimated prevalence. The selection criteria for this study were women: (1) married (2) not pregnant (3) had not undergone a hysterectomy or conization and (4) not mental impaired. Only married women were selected because under cultural/ethical norms of Viet Nam, it was not feasible to implement pelvic examinations and Pap test among unmarried women.

In each city, eligible women were randomly selected from the list of married women aged from 18–69 year old provided by the Women Union. These selected women were visited by staffs of Women Union at their home to explain about the objectives of the study as well as to obtain a written consent form for their participation in this study. The response rates in both rounds were very high due to the benefits of attending this study (i.e., free HPV test, free PAP smear), however due to limited funding for testing, we were able to select only 750 women/each city in round 1 and 1000 women/each city in round 2.

Information and specimen collection used the following steps. First, a personal interview was done to collect information on socio-demographic variables, obstetric/gynecologic history, and sexual lifestyle. After interviewing, each participant was scheduled for a pelvic examination carried out by a gynaecologist. For Pap sample, a wooden spatula was gently used to collect cells from the ectocervix, the cells then were place on a glass slide preserved with a fixative, stained. Another wooden spatula was used to collect HPV sample. Both HPV and Pap samples were sent to the laboratory of Vietnam Institute of Dermatology on the same day of collection.

The HPV genotyping protocol includes 4 steps. The first step is to receive and treat samples using cotton swab and storage solution. The second step is to extract DNA HPV using DNA extraction kit based on phenol/chloroform. The third step is to use the real-time PCR kit to detect HPV DNA using TaqMan probe. In step 4, genotype by reverse dot blot (RDB), was applied for all the HPV postive samples. RDB was a simple and fast typing procedure for detecting 37 mucosotropic HPV types with high sensitivity/specificity [9].

The study protocol was submitted to the Hanoi school of Public Health IRB, registered with U.S. Dept. of Health and Human Services - IORG number 0003239, FWA number FWA00009326. The protocol was reviewed and cleared by this ethical committee (Ethical Approval Number 013/2010/YTCC-HD3).

## Results

### Characteristics of population

Table 1 presents the characteristics of study subjects. The median age of women participated in this study was 40 year old (ranged from 18 to 65 year old) The majority of women were living with their husband. *Prevalence of cervical infection with HPV type 16 and/or 18.*

**Table 1:** Characteristics of study sample (Total sample = 4500)

**Characteristics**		**N (%)**
Age group	<30	611 (13.6)
	30-39	1401 (31.1)
	40-49	1531 (34.0)
	< = 50	957 (21.3)
Highest education attained	Primary	682 (15.2)
	Secondary	1363 (30.3)
	High school	1357 (30.2)
	Higher than high school	1088 (24.2)
Occupation	Government officers	843 (18.7)
	Workers/handicraft	434 (9.6)
	Small trade	1079 (24.0)
	Un-employed/House-wife/Retired	1554 (34.5)
	Other	590 (13.2)
Marital status	Live with husband	4087 (90.8)
	Separated/Divorced	261 (5.8)
	Widower	152 (3.4)

**Figure 1:**
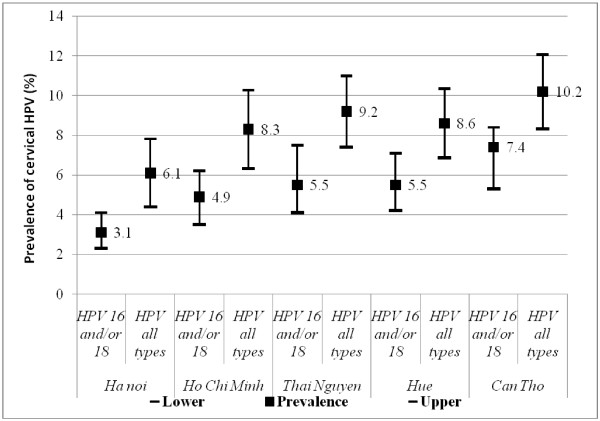
Prevalence of HPV 16 and/or 18 in 5 studied cities.

Figure 1 presents the prevalence of cervical infection with HPV type 16 and/or type 18 along with the prevalence of infection with all type of HPV. The prevalence of cervical infection with HPV type 16 and/or 18 among married women ranged from 3.1% in Hanoi to 7.4% in Can Tho. A strong correlation between the prevalence of infection with HPV type 16 and/or 18 and the overall prevalence of HPV can be observed; city with higher overall prevalence of HPV infection was also the place with higher prevalence of infection with HPV type 16 and/or type 18.

**Figure 2:**
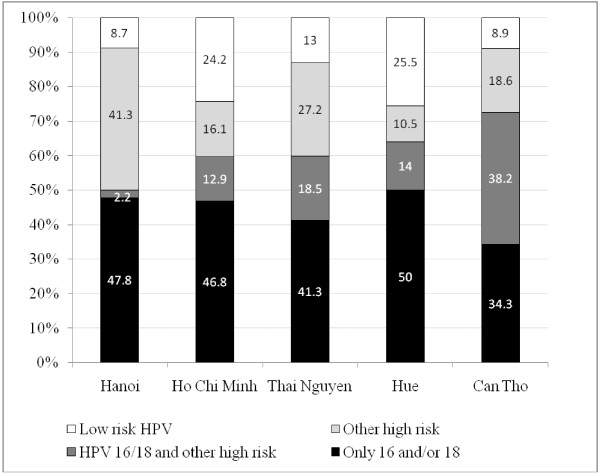
Distribution of infection with HPV 16 and/or 18 and other HPV types among 5 cities.

Figure 2 presents the distribution of infection with HPV 16 and/or18 and other HPV types among the positive cases. The proportion of infection with only HPV 16 and/or 18 among positive cases were highest in Hue (50%) and lowest in Can Tho (34.3%). So at maximum, half of the positive cases were infected with only HPV 16 and/or 18, and the rest were infected with other types of HPV. The total proportion of infection with other high risk type of HPV is the sum of the proportion of infection with other high risk HPV types (i.e., light grayshade in the graph) and that of infection with HPV 16 and/or 18 and other high risk (dark gray shade in the graph); it ranged from 24.5% in Hue to 56.8% in Can Tho.

**Figure 3:**
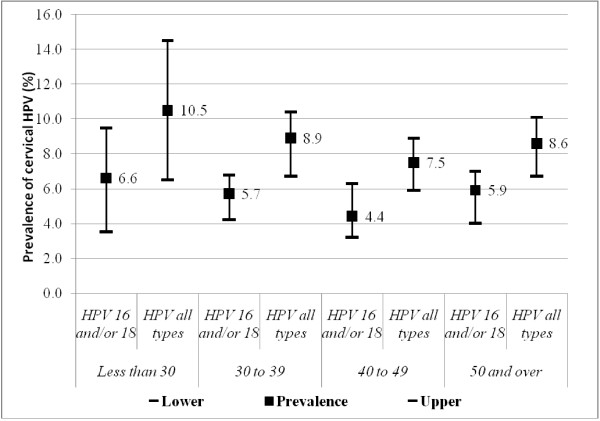
Prevalence of HPV 16 and/or 18 and all type HPV infection by age groups.

Figure 3 shows the prevalence of infection with HPV 16 and/or 18 and infection with all HPV types by 4 age groups (i.e., using aggregated data for women from all cities). These prevalence are different among stratified age group (highest among young married women, aged less than 30 and lowest among women aged from 40 to 49 year old); however, Chi-square test indicated that these differences were not significant (p for HPV 16/and or 18 was 0.10 and for all HPV types was 0.14) .

### Other types of high risk HPV

This study detected a total of 24 HPV types; of these, 8 types were low risk (HPV6, 11, 42, 43, 61, 70, 71 and 81) and 16 types were high risk (HPV16, 18, 31, 33, 35, 39, 45, 51, 52, 53, 56, 58, 59, 62, 66, and 68). Table 2 show 5 most common HPV types in each studied city.

**Table 2:** The five most common HPV types among 5 cities

**Hanoi**	**HCM**	**Thai Nguyen**	**Hue**	**Can Tho**
HPV 16 (1.7%)	HPV 18 (4.4%)	HPV 16 (3.8%)	HPV 18 (3.6%)	HPV 18 (4.9%)
HPV 18 (1.5%)	HPV 16 (1.5%)	HPV 18 (2.5%)	HPV 16 (2.7%)	HPV 16 (4.8%)
HPV 58 (1.2%)	HPV 58 (0.9%)	HPV 58 (1.7%)	HPV 58 (1.1%)	HPV 58 (2.2%)
HPV 45 (0.5%)	HPV 35 (0.5%)	HPV 59 (0.8%)	HPV 35 (0.3%)	HPV 52 (1.3%)
HPV 53 (0.3%)	HPV 52 (0.5%)	HPV 33 (0.6%)	HPV 33 (0.2%)	HPV 35 (0.8%)

In addition to HPV high risk type 16 and 18, there were other 14 HPV high risk types. The 5 most common other HPV high risk types among the whole sample were HPV58 (1.47%), HPV52 (0.49%), HPV35 (0.44%), HPV45 (0.38%) and HPV59 (0.38%).

### Abnormal Pap smear and HPV positive

Of 4500 women participated in this study, 177 women (3.9%) had abnormal Pap smear results. Specifically, 104 women had ASCUS (atypical cells of undetermined significance), 39 had AGUS (atypical glandular cells of undetermined significance), 26 had LSIL (low grade squamous intraepithelial lesion), and 8 had HSIL (high grade squamous intraepithelial lesion). Table 3 present the distribution of HPV (+) and HPV 16/and or 18 (+) among the abnormal Pap results, about 37.2% women with abnormal Pap results had HPV positive and 16.9% of them infected with type 16 and or 18.

**Table 3:** Abnormal Pap smear results and HPV positive

**PAP results**	**Total cases**	**Total cases with HPV (+)**	**Cases with 16/18 (+)**
AGUS	39	4 (10.3%)	3 (7.0%)
ASCUS	104	46 (44.2%)	12 (11.5%)
LGSIL	26	13 (50.0%)	12 (46.2%)
HGSIL	8	3 (37.5%)	3 (37.5%)
Total	177	66 (37.2%)	30 (16.9%)

### Awareness of HPV and HPV vaccines

**Figure 4:**
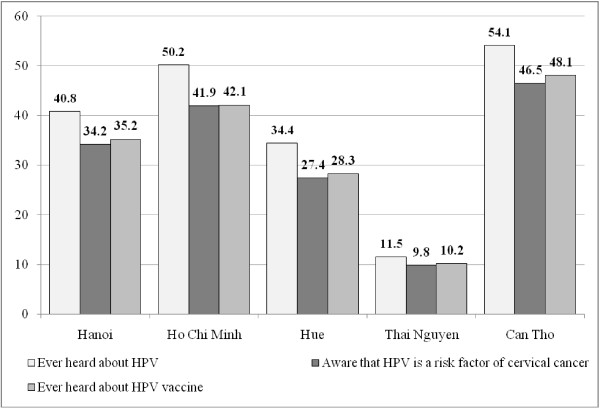
Awareness of HPV and HPV vaccines across 5 cities.

Figure 4 presents information about the awareness of HPV and HPV vaccines among married women in 5 cities. In the overall sample size, about 37.4% women ever heard about HPV, 31.2% knew about HPV vaccine and 32.1% aware that HPV is a risk factors of cervical cancer. The level awareness varies significant across 5 cities. In wealthier cities like Hanoi or Ho Chi Minh, the level of awareness was much higher.

## Discussion

The prevalence of cervical infection with HPV type 16 and/or 18 among married women in this study ranged from 3.1% to 7.4%. This prevalence was highest in Can Tho, this figure was also consistent with the fact that among 5 cities, Can Tho had highest rate of cervical cancer [10]. Most of HPV positive cases in this study were infected with HPV type 16 or 18. So this finding was similar to previous studies in Vietnam and other countries [8,11,12].

Results also indicated that among cases with abnormal Pap results, 16.9% of them infected with HPV 16 and/or 18; however, the sample size of women with abnormal Pap smear was not sufficient enough to examine the distribution of HPV 16 and/or 18 among abnormal Pap cases. Data about the composition of HPVs present in cervical cancer in Vietnam was also not available from previous research. It is important to note that the distribution of HPV type 16/and or 18 among general population would be different to that among cancer cases. So the distribution of HPV 16/and or 18 among general married women in this study cannot be considered as a proxy for that in cervical cancer and it was a limitation of this study.

In addition to HPV 16 and 18, this study also reported 14 other types of high risk HPV, most notably was HPV type 58, 52, 35 and 45. Other studies also reported that HPV 58 was among the most common types found in cervical cancer specimens in China, Thailand and The Philippines [13]. A recent meta analysis about HPV positive and cervical cancer had showed that HPV45 (in Africa and South/Central America) and HPV 58 (in Eastern Asia) accounted for important proportions of HPV-positive CIN2 and CIN3 [6]. HPV58 and HPV45 were also common high risk type in Vietnam. The current available vaccines may have cross-protective effects against 4 types, HPV-33, HPV-31, HPV-45, and HPV-51, in addition to HPV 16 and 18 [14] but no evidence about the protective effects with HPV 58 has been reported. Thus, while it is relevant to implement an HPV vaccine campaign in Viet Nam due to the high prevalence of infection with HPV 16 and/or 18, it is also important to inform the women who receive the vaccines that they are not protected against all high risk HPV types and that they still need cervical cancer screening. A recent study also showed that two doses of the HPV16/18 vaccine, and maybe even one dose, are as protective as three doses [15]. As the current three-dose regiments for HPV vaccines are expensive and difficult to complete, the vaccine campaign in Vietnam may consider to offer the two dose regiment instead.

Vietnam still applies the opportunistic cytology-based screening model and this model has failed to have an impact on the overall incidence of cervical cancer in Vietnam. A new potential model “screen and treat” using either HPV DNA testing or VIA (visual inspection with acetic acid) followed by treatment with cryotherapy (freezing) proposed by the Alliance for Cervical Cancer Prevention may be applied to the Vietnam context [16].

Results showed that about 37.4 women ever heard about HPV and 31.2% knew about HPV vaccine. A previous study among women in the United states reported that the level of awareness of HPV and of HPV vaccine were 84.3% and 78.9% [17]. More comparable, a study among the rural folks in Penang Malaysia estimated that about 42.2% women ever heard about HPV vaccine [18]. So compared to other countries, the level of HPV and HPV vaccine awareness were limited, in order to make the vaccine campaign become more effective, Vietnam need to have more mass media campaign to provide more information about HPV and HPV vaccines.

Strict protocols to avoid biases were followed in this study: women were randomly chosen, all clinical examination and specimen collections were done by qualified gynaecologists and all the samples were examined by a nationally qualified laboratory. The detection of HPV positivity using real time PCR methods and the genotyping of HPV type using reverse dot blot method in this study also provided more precise results compared to the Hybrid Capture Tube Method applied in previous studies [19]. One also may question while the target of HPV vaccination should be adolescents and this study captured mostly women over 30 year old.

This is a limitation of this study because we were not able to invite unmarried women to attend study with pelvic examinations and cervical sampling collect due to the strict cultural/ethical norms in Vietnam so the results did not cover a subgroup a the young population already sexually active but not yet married. It can also be argued that probably HPV detected in older women would have more likely to be present for a longer period, at higher risk of persistent infection and higher risk to develop lesions and cancer so the data presented in this study would be very useful for the cervical screening programming in Vietnam.

## Conclusion

In conclusion, a high prevalence of HPV infection, especially high risk types, was observed in this study. As HPV infection has a high correlation with cervical cancer, this study emphasizes the need for both primary prevention of cervical cancer with HPV vaccines as well as secondary prevention with screening. Currently, in Vietnam HPV vaccines is not yet offered in routine EPI program and the cervical screening program still applies the opportunistic cytology-based screening model. Policy-makers in Viet Nam should consider making HPV vaccines and screening become organized routine practices for cervical cancer prevention.

## Competing interests

The authors declare that they have no competing interests.

## Authors’ contribution

LV designed, carried out the survey, coordinated the sequence alignment and drafted the manuscript. HL and DB participated in the design of the study and performed the statistical analysis. All authors read and approved the final manuscript.

## Pre-publication history

The pre-publication history for this paper can be accessed here:

http://www.biomedcentral.com/1471-2407/13/53/prepub
